# Development of an Optimized Drying Process for the Recovery of Bioactive Compounds from the Autumn Fruits of *Berberis vulgaris* L. and *Crataegus monogyna* Jacq.

**DOI:** 10.3390/antiox10101579

**Published:** 2021-10-07

**Authors:** Cadmiel Moldovan, Oleg Frumuzachi, Mihai Babotă, Luigi Menghini, Stefania Cesa, Alexandru Gavan, Cristian R. Sisea, Corneliu Tanase, Maria Inês Dias, Carla Pereira, Isabel C.F.R. Ferreira, Gianina Crișan, Andrei Mocan, Lillian Barros

**Affiliations:** 1 Faculty of Pharmacy, “Iuliu Hațieganu” University of Medicine and Pharmacy, 8 Victor Babeș Street, 400012 Cluj-Napoca, Romania; cadmiel.moldovan@umfcluj.ro (C.M.); oleg.frumuzachi@elearn.umfcluj.ro (O.F.); mihai.babota@umfcluj.ro (M.B.); gavan.alexandru@umfcluj.ro (A.G.); gcrisan@umfcluj.ro (G.C.); 2 Department of Pharmacy, Botanic Garden “Giardino dei Semplici”, Università Degli Studi “Gabriele d’Annunzio”, Via dei Vestini 31, 66100 Chieti, Italy; lmenghini@unich.it; 3 Department of Drug Chemistry and Technologies, University “Sapienza” of Rome, P.le Aldo Moro 5, 00185 Rome, Italy; stefania.cesa@uniroma1.it; 4 Laboratory of Chromatography, Institute of Advanced Horticulture Research of Transylvania, University of Agricultural Sciences and Veterinary Medicine, 400372 Cluj-Napoca, Romania; cristian.sisea@usamvcluj.ro; 5 Faculty of Horticulture, University of Agricultural Sciences and Veterinary Medicine Cluj-Napoca, 400372 Cluj-Napoca, Romania; 6 Faculty of Pharmacy, “George Emil Palade” University of Medicine, Pharmacy, Science and Technology, 38 Gheorghe Marinescu Street, 540142 Târgu-Mureș, Romania; corneliu.tanase@umfst.ro; 7 Centro de Investigação de Montanha (CIMO), Instituto Politécnico de Bragança, Campus de Santa Apolónia, 5300-253 Bragança, Portugal; maria.ines@ipb.pt (M.I.D.); carlap@ipb.pt (C.P.); iferreira@ipb.pt (I.C.F.R.F.); lillian@ipb.pt (L.B.)

**Keywords:** drying process optimization, barberry, hawthorn, antioxidant activity, autumn fruits

## Abstract

Hot air drying has proven to be an efficient method to preserve specific edible plant materials with medicinal properties. This is a process involving chemical, physical, and biological changes in plant matrices. Understanding these processes will lead to an improvement in the yields of bioactive compounds. This study aims to optimize the drying process of two species’ fruits used in folk medicine, *Berberis vulgaris* and *Crataegus monogyna*. The optimized extracts’ antioxidant capacity was assessed using various assays, with the barberry extract showing very good activity (50.85, 30.98, and 302.45 mg TE/g dw for DPPH, TEAC, and FRAP assays, respectively). Both species exerted good fungal α-glucosidase inhibitory activity (IC_50_ = 0.34 and 0.56 mg/mL, respectively) but no activity on mammalian α-glucosidase. Additionally, this study identified and quantified the main bioactive compounds. The results presented herein are a breakthrough in industrializing this drying process. Additional studies are necessary to mechanistically understand the drying process involved in these plant materials.

## 1. Introduction

Medicinal plants have traditionally been used since the earliest times for the treatment of a wide range of ailments. Many of them are appreciated not only for their nutritional value but also for their organoleptic characteristics, for which they are used in dishes [1]. One of the most used methods for plant conservation, whether for medicinal or culinary purposes, is the drying process. Although drying is at first glance a process that involves the removal of water from the plant material, there are many physical, chemical, and biological transformations that occur during this process. Moreover, through drying, the water activity and moisture content of the foods are reduced, so the growth of microorganisms in the foods is largely prevented/postponed [2].

Numerous drying techniques have been developed and used for the dehydration of vegetal products over the years. The most relevant techniques involved in vegetal product drying are hot air drying, spray drying, freeze-drying, and osmotic dehydration [3]. Hot air drying is a traditional drying method in fruit dehydration. Through the hot air drying process, the vegetables are dried to enhance storage stability, minimize packaging requirements, and reduce transport cost. Currently, the hot air drying process is the main drying method used in vegetable dehydration. In this process, the pretreated fruits are subjected to hot airflow of 50–90 °C. Heat can be transferred from hot air to the vegetables, and when the heat is absorbed by the materials, two types of moisture diffusion occur. One process is external diffusion, in which moisture moves from the material surface to the dry medium. The other process is internal diffusion, in which the internal moisture moves to the material surface. These two diffusion processes develop at the same time until the moisture content decreases to the level at which the materials can be stored safely [4]. In this study, the main process in vegetable dehydration, the air drying technique, was chosen, which is a well-established technology owing to the availability of the necessary equipment and facilities.

Significant evidence has been gathered that indicates the key role of reactive oxygen species (ROS) and other oxidants in causing numerous ailments and diseases [5]. This proof has brought the attention of scientists to an appreciation of antioxidants for the prevention and treatment of diseases and maintenance of human health. The human body has an inherent antioxidative mechanism and many of the biological functions such as the anti-carcinogenic, anti-mutagenic, and anti-aging responses originate from this property. Antioxidants stabilize or deactivate free radicals, often before they attack targets within biological cells. Recently, interest in naturally occurring antioxidants has considerably increased for use in food, cosmetics, and pharmaceutical products because they possess activities that provide enormous potential to correct imbalances [6].

The genus *Berberis* consists of around 500 species of widely distributed 1–5 m tall evergreen shrubs [7]. The different plants of the genus *Berberis* can be found in many regions of the world, although their main distribution is in the Northern Hemisphere, more exactly, in the Himalayan region. The first mention of these plants was made in medieval writings. In the first century A.C. the Greeks were familiar with a *Berberis* extract, which they named “Indian Lyceum”, while the Arabs termed it “Honduras” [8]. Some *Berberis* sp. are commonly used in folk medicine for the treatment of gastric and duodenal ulcers, chronic diarrhea, and rheumatic conditions of the joints. Notably, despite the fact that the *Berberis* genus is known for its specific, well-documented alkaloids, berberine and berbamine, these compounds are found only in the stems and roots [9].

Among the many species of this genus, *Berberis vulgaris* L. is a well-known shrub native to central and southern Europe, western Asia, and northwest Africa. It is known under the names of “common barberry”, “European barberry”, “jaundice berry”, and “ambarbaris”. The biggest producer of *B. vulgaris* in the world is Iran, with ~11,000 hectares of land under cultivation. Moreover, ~10,000 tons of dried *B. vulgaris* fruits are produced per year in Iran [10]. The fruit, known as barberry, is an oblong red berry 7–10 mm in length and 3–5 mm in width, which ripens usually at the end of summer/beginning of autumn. Historical testimonies revealed that drinking barberry juice can ameliorate fever, thirst, and even inflammation [8]. Additionally, the derivatives of barberry are used to produce traditional products such as marmalade, jam, carbonated drinks, and sauces. The anthocyanins extracted from the fruits are industrially used as coloring agents for the preparation of food products, such as juices [10].

The medicinal use of the fruits of *B. vulgaris* in the literature is lacking, but there are many other traditional uses cited for the other parts of the plant (e.g., roots and bark), but they are not the object of this study.

Regarding the chemical composition of barberry fruits, the scientific data shows that they contain little or no alkaloids. However, they contain a great amount of phenolics, gum, pectin, oleoresins, and organic acids [11].

Several pharmacological effects have been demonstrated for the barberry fruit extract. Among these are cytoprotective and antioxidant actions [12] and anticholinergic and antihistaminergic effects [11].

Hawthorn is a common name of all species in the genus *Crataegus*. The generic name, *Crataegus*, comes from the Greek *kράτος*, meaning hard or strong, referring to the plant’s wood. Nowadays, it is known that almost 20 species of hawthorn are used as drug materials or herbal medicines worldwide. Some of them are listed in the pharmacopoeias of various countries. Their use for the treatment of cardiovascular disease began in the late 1800s. In traditional Chinese medicine (TCM), hawthorn fruits are used to stimulate digestion and to improve blood circulation. Additionally, in European folk medicine, hawthorn is used as a cardiotonic, astringent, anti-spasmodic, and diuretic agent [13,14].

The red fruit (frequently called “haw”) of *Crataegus monogyna* Jacq. (common hawthorn), which ripens generally in the middle of autumn, is traditionally used for different culinary purposes, such as the preparation of jellies, jams, and syrups [14,15].

Moreover, the fruits from *C. monogyna* are mentioned in different pharmacopeias worldwide such as the European Pharmacopeia or British Pharmacopeia [16]. In Romania, the name of the species is “Păducel” or “Mărăcine”. The pharmacological proprieties of hawthorn fruit extracts are usually attributed to proanthocyanidins and other glycosylated derivatives of flavonoids [17]. Various studies have aimed to assess the in vivo or in vitro activities of these compounds towards cardiovascular, digestive, or reproductive ailments. Furthermore, it was demonstrated that extracts from *C. monogyna* fruits can ameliorate inflammation, exert antimicrobial activity, and can be used as an adjuvant in cancer radiotherapy.

Given these important characteristics, the aims of this study were to assess the effects of drying conditions on two traditionally used fruits in Romania (barberry (*B. vulgaris*) and hawthorn (*C. monogyna*)) and to select the proper conditions with respect to time of drying and air temperature.

## 2. Materials and Methods

### 2.1. Chemicals

Acetone, Hydrochloric acid (37%), Folin–Ciocâlteu reagent, methanol and ethanol were purchased from Merck (Darmstadt, Germany). 2,2-diphenyl-1-(2,4,6-trinitro-phenyl) hydrazine (DPPH), 2,5,7,8-tetramethylchromane-2-carboxylic acid (Trolox) (97%), ferric chloride, sodium carbonate, 2,4,6-Tris(2-pyridyl)-S-triazine (TPTZ) (≥99%), dimethyl sulfoxide (DMSO) (≥99%), 3,4-Dihydroxy-L-phenylalanine (L-DOPA) (≥98%), 6-hydroxy-diammonium 2,2′-azinobis(3-ethylbenzothiazoline-6-sulfonate) (ABTS) (>98%), mushroom tyrosinase, phosphate buffer, and kojic acid were purchased from Sigma (Sigma Aldrich Chemie GmbH, Schnelldorf, Germany). Aluminum chloride (≥98%) was acquired from Carl Roth (Karlsruhe, Germany). Water was of Milli-Q-quality. All solvents were of LC grade and all reagents were of analytical grade.

### 2.2. Sample Preparation

The plant materials obtained from different locations in Abruzzo Region, Italy and Cluj County, Romania were refrigerated at 5 °C to prevent additional damage of samples. Fruits with undesirable aspects (unripe and burst characteristics) were removed. After identification, fruit samples were stored in the Botany Department’s Herbarium, Faculty of Pharmacy, Cluj-Napoca.

### 2.3. Drying Procedure

The first step of the procedure was represented by some preliminary experiments that generated data (not shown) on the critical process parameters and quality attributes of the plant-derived products (e.g., drying time, air flow, air temperature, distance between fruits samples). Subsequently, the drying strategy was developed. The software MODDE Pro 11.0 (Sartorius, Sweden) was used to develop and analyze the design of the experiments and to define the processes and the optimal drying parameters.

For the drying procedure, an oven dryer (Excalibur Food Dehydrator, Sacramento, CA, USA) was used at three different temperature levels (50, 60, and 70 °C). The relative air humidity and the ambient air temperature of the environment where the dryer was operating were 40–50% and 16–22 °C, respectively. The air flow temperature was accurately monitored over time. To record weight changes in the samples during drying, a balance (Adam PW254, Adam Equipment Co. Ltd., Milton Keynes, UK) with a sensitivity of 10 mg was used. Samples’ weight was checked at 4, 8, 12, 18, 24, 36, and 48 h. A steady state condition was achieved in the dryer system at the beginning of the experiments. The fruits (5 g/sample) were spread uniformly on a perforated tray and subsequently inserted into a preheated oven.

### 2.4. The Procedure for Extraction

The following method was employed for the extraction: dried fruits’ samples were ground and homogenised using a mill, namely Retsch Grindomix GM200 (Haan, Germany). Fifty mL of 70% (*v*/*v*) ethanol were added to the previously obtained powder and for homogenization, a vortex apparatus (Velp Scientifica Classic, Bohemia, NY, USA) was used. The mixture was ultrasonicated for 30 min at 50 °C and filtered using a water vacuum filter. The solution was made up with EtOH 70% to a final volume of 50 mL.

The reconstitution of extracts was done as follows: for total bioactive compound quantification and for antioxidant activity, the dry weight extract was redissolved in EtOH 70% and for the enzymatic assays the dry weight extract was redissolved in water with 5% DMSO.

### 2.5. Analysis of Phenolic Compounds

The polyphenolic profile was determined by means of an LC-DAD-ESI/MS^n^ (Dionex Ultimate 3000 UPLC, Thermo Scientific, San Jose, CA, USA) using the previously described method by Bessada et al. (2016) [18]. The extracts were redissolved with ethanol:water (80:20, *v*/*v*) at a concentration of 50 mg/mL. Online detection was carried out by DAD (preferred wavelengths of 280, 330, and 370 nm) coupled with a mass spectrometer (MS). MS detection was carried out using a Linear Ion Trap LTQ XL MS acquired from Thermo Finnigan (San Jose, CA, USA) in negative mode with an ESI source.

Based on polyphenol’s chromatographic behavior and UV-vis and mass spectra, it was possible to identify the compounds comparing the data obtained with standard compounds, when available. The acquisition of data was performed with an Xcalibur^®^ data system (Thermo Finnigan, San Jose, CA, USA). Based on the UV-vis signal, a 7-level calibration curve for each available phenolic compound was depicted in order to perform quantitative analysis. If a commercial standard compound was not available, the quantification was carried out using the calibration curve of the most similar available standard. Finally, the results were expressed as mg/g of extract.

### 2.6. Quantitative Determination Total Flavonoid Content (TFC) and Total Phenolic Content (TPC)

The total phenolic content (TPC) of the extracts was determined using Folin-Ciocâlteu method, previously described by Mocan et al. (2016) [19]. Briefly, 100 μL of Folin-Ciocâlteu reagent (diluted 1:10 with distilled water) were mixed with 20 μL of each sample in a 96 well plate and incubated at room temperature. A solution of sodium carbonate (7.5% *w*/*v*, 80 μL) was added after 3 min to the wells and the mixture was incubated once again. After 30 min, the absorbance of the mixture was measured at 760 nm, using a SPECTROstar Nano microplate reader (BMG Labtech, Offenburg, Germany). Gallic acid was used as a reference standard and the TPC results of the extracts were expressed as milligrams of gallic acid equivalents (GAE)/gram of dry weight (dw) of raw fruits (mg GAE/g dw) or/gram of dry weight of optimized lyophilized extract (mg GAE/g dw).

The crude extracts’ total flavonoid content (TFC) was assessed using a method previously reported by Mocan et al. (2017) [20]. Briefly, to 100 μL of sample were added 100 μL of 2% AlCl_3_ aqueous solution in a 96 well plate. After 15 min of incubation in a dark place and at room temperature, the absorbance was read at 420 nm using a microplate reader, namely SPECTROstar Nano (BMG Labtech, Offenburg, Germany). Quercetin was used as a reference standard and the TFC results of the extracts were expressed as milligrams of quercetin equivalents (QE)/gram of dry weight (dw).

### 2.7. Antioxidant Capacity Assays

ABTS radical cation scavenging activity (TEAC), DPPH radical scavenging activity (DPPH) and ferric-reduction antioxidant power (FRAP) were used to assess the antiradical activity of the samples. A thiobarbituric acid reactive substances (TBARS) assay was used to analyze the inhibition of the lipid peroxidation process. Furthermore, the oxidative hemolysis inhibition assay (OxHLIA) was applied in order to determine the relevance of the in vivo inhibition of the free radicals.

#### 2.7.1. DPPH Radical Scavenging Activity Assay (DPPH)

DPPH radical scavenging activity assay was assessed by a method described earlier by Moldovan et al. (2021) [21] with some modifications. Briefly, 30 μL of sample solution and 0.004% DPPH radical solution (5 mg of DPPH diluted in 5 mL of absolute MeOH, followed by a 25-fold dilution of 1 mL DPPH solution) was incubated in a dark place at around 20–22 °C. After 30 min, the absorbance of the samples was measured at 515 nm. DPPH radical scavenging activity was expressed in milligrams of trolox equivalents (TE)/gram of dry weight mg TE/g dw).

#### 2.7.2. Trolox Equivalent Antioxidant Capacity (TEAC) Assay

A TEAC assay was assessed by a method previously described by Mocan et al. (2014) [22]. In brief, 20 μL of sample and 200 μL of radical solution, consisting of ABTS stock solution: K_2_S_2_O_8_ stock solution (1:1) diluted 1:5 with water, were incubated for 6 min. The scavenge activity of antioxidants against ABTS^+^ radical was measured at 760 nm. The antioxidant activity according to TEAC assay was expressed as milligrams of trolox equivalents (TE) per gram of dry weight (dw).

#### 2.7.3. Ferric Reducing Antioxidant Power Assay (FRAP)

A FRAP assay was conducted as previously reported by Mocan et al. (2018) [23] with some modifications. In brief, a FRAP reagent was made by mixing 300 mM acetate buffer (pH 3.6), 10 mM TPTZ reagent (31.23 mg TPTZ diluted in 10 mL of 40 mM HCl), and 20 mM FeCl_3_ solution at a ratio of 10:1:1 (*v*:*v*:*v*). A sample solution amounting to 25 μL and 175 μL of FRAP reagent were mixed in a 96 well plate and incubated in a dark place at 20–22 °C. After 30 min, the absorbance of the samples was measured at 593 nm. FRAP antioxidant activity was expressed as milligrams of trolox equivalents (TE)/gram of dry weight (mg TE/g dw).

#### 2.7.4. Thiobarbituric Acid Reactive Substances Assay (TBARS)

TBARS assay was conducted as previously described by Souilem et al. (2017) [24] using porcine brain homogenates. Following this method, the absorbance of malondialdehyde-2-thiobarbituric acid [MDA-TBA] complex found in the supernatant at the end of the assay was measured at 532 nm. The concentration of the sample that inhibited lipid peroxidation in proportion of 50% was taken as the EC_50_ value, expressed in μg/mL. Trolox was used as a positive control.

#### 2.7.5. Oxidative Hemolysis Inhibition Assay (OxHLIA)

In order to obtain solutions with a final concentration ranging from 12.5 to 125 μg/mL, 100 mg of each extract were dissolved in phosphate-buffered saline (PBS). Oxidative hemolysis was measured by a method described by Takebayashi et al. (2012), using ovine erythrocyte solution [25]. Different extract concentrations and their afferent Ht_50_ values were used to infer the concentration of the extract capable of 60 min Δt hemolysis retardation [26]. Thus, the extract concentrations required to keep 50% of the erythrocyte population intact for 60 min were expressed as EC_50_ values (μg/mL), with Trolox being used as a positive control.

### 2.8. Cytotoxic Activity

In order to asses cytotoxic activity, concentrations ranging from 6.25 to 400 µg/mL were obtained from re-dissolving the extracts in distilled water and further dilution. Evaluation of the cytotoxic properties of the extracts was done on human tumor cell lines (breast adenocarcinoma—MCF-7, non-small cell lung cancer—NCI-H460, cervical carcinoma—HeLa, and hepatocellular carcinoma—HepG2) and a non-tumour cell line—PLP2—using a method described by Abreu et al. (2011) [27].

Sulforhodamine B assay was performed using a method described by Barros et al. (2013) [28] using Ellipticine as positive control, and a negative control provided by each suspension of cells. The results were expressed as GI_50_ values, which represent the concentrations at which 50% of cell proliferation was inhibited.

### 2.9. Anti-Inflammatory Activity

Anti-inflammatory activity was assessed by a method previously described [24]. In brief, extracts were re-dissolved and diluted in distilled water to concentrations that ranged from 6.25 to 400 µg/mL. Griess Reagent System (GRS) kit was applied to measure the nitric oxide using a mouse macrophage-like cell line RAW 264.7. The final absorption was read at 515 nm using an ELx800 microplate reader (Bio-Tek Instruments, Inc; Winooski, VT, USA). The final results were expressed as IC_50_ values. Dexamethasone (Dex) was used as a positive control, while the negative control contained no bacterial lipopolysaccharides (LPS).

### 2.10. Inhibition of Fungal α-Glucosidase

Inhibition of fungal α-glucosidase (AGLU) was carried out by a method described by Sakna et al. (2019) [29] with slight modifications. In brief, fifty microliters of extract solution (sequential dilutions) and fifty microliters of the enzyme solution (at a concentration of 2 U/mL) diluted in phosphate buffer (0.1 M, pH 6.8) were mixed in a 96-well plate and incubated in a place protected from light at a temperature of 37 °C. After 10 min, 50 μL of α-pNPG solution (2.5 mM), also prepared in phosphate buffer (0.1 M, pH 6.8), were added to the mixture and incubated at 37 °C, in a place protected from light. After another 10 min, the absorbance was measured at 405 nm spectrophotometrically.

AGLU activity was calculated according to the following formula:

(1) $$\mathrm{Inhibition}\left(\%\right)=\frac{\left(A-B\right)-\left(C-D\right)}{\left(A-B\right)}\times 100$$

where A is the absorbance of the control and B is the absorbance of the blank control. C and D are the absorbances of the sample and blank sample. The α-glucosidase inhibitory activity was expressed as an IC_50_ value.

### 2.11. Rat α-Glucosidase Inhibitory Assay

For the rat α-glucosidase inhibitory assay, rat intestinal acetone powder dissolved in phosphate buffer (0.1 M, pH 6.9) at a ratio of 50:1 (*w*:*v*), was centrifuged for 10 min (4000 rpm). Enzyme solution for rat α-glucosidase inhibition was prepared using a five-fold diluted supernatant with phosphate buffer, the rest of the assay procedure being performed in the same method as that for the inhibition of fungal α-glucosidase [29].

### 2.12. Statistical Analysis

The experiments were carried out in triplicate and the data are expressed as mean values ± SD for each sample. Moreover, data were evaluated by one-way analysis of variance in order to identify significant differences between values. Differences described by a *p* value of <0.05 were considered significant. The statistical significance of differences and the statistical correlation between data were calculated using SPSS 16.0 (Armonk, NY, USA) for Windows.

## 3. Results and Discussion

### 3.1. Drying Process Optimization

**Figure 1:**
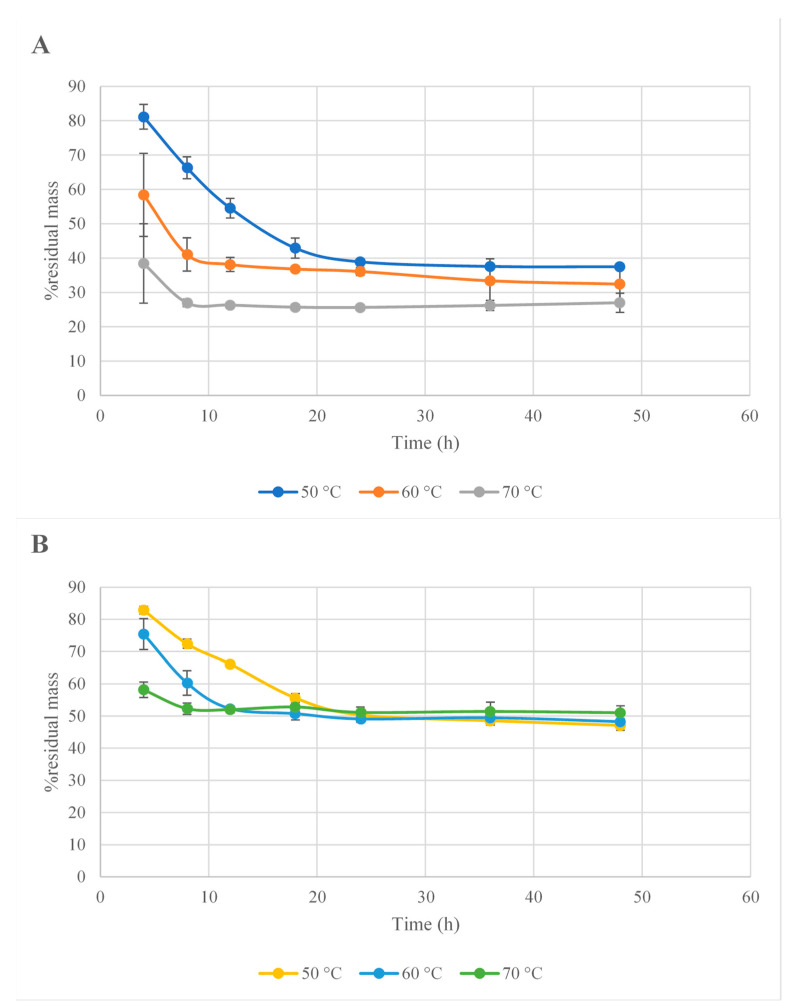
Residual mass percentage versus drying time for temperatures of 50, 60, and 70 °C in *B. vulagris* (**A**) and *C. monogyna* (**B**).

**Figure 2:**
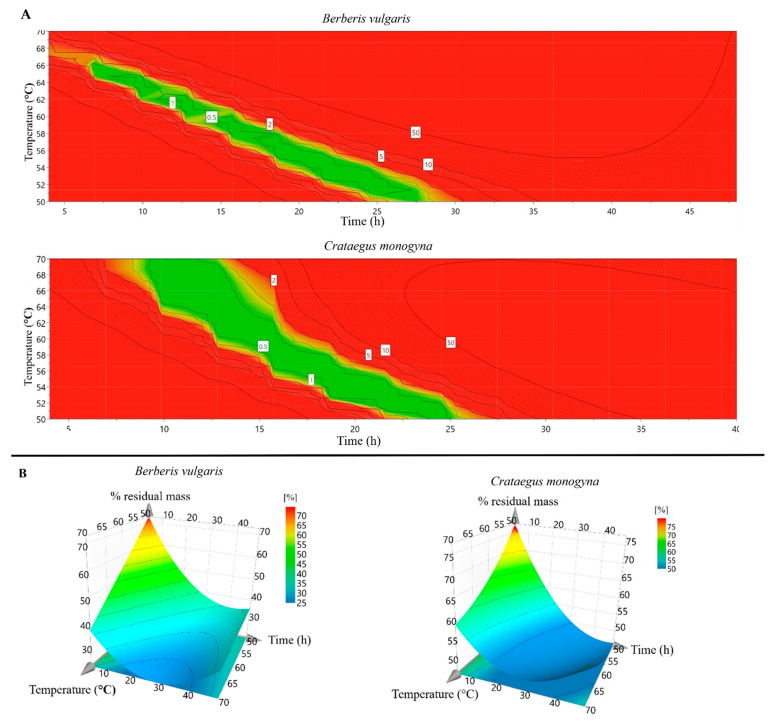
(**A**) Optimal temperature conditions represented in design spaces as a function of time for drying processes, (**B**) 3D-response surface plots: The interdependence between critical drying process parameters (temperature and time of drying) with the percent of residual mass for *B. vulgaris* and *C. monogyna* samples.

The drying process of plant materials was analyzed and optimized using a specialized software, MODDE Pro, v. 11.0. The temperature of the air flow plays a crucial role in the process, as its increase or decrease can lower or higher the drying time, respectively, as can be seen in Figure 1 and Figure 2. On the other hand, it is well known that the temperature can alter the structure of certain plant bioactive compounds [30]. Hence, given these implications, the drying temperature was lowered so that a lower amount of bioactive compounds would be destroyed. However, as the scope of this research was to extend the process to an industrial scale, we needed to find a balance between the temperature and the time required for the whole process. Consequently, the optimal temperature considered was 60 °C. The drying parameters were integrated into the software and the optimal time of fruit dried at 60 °C was calculated.

Based on the developed experimental model, the optimal time required for drying the barberry fruits (*B. vulgaris*) at 60 °C was calculated and it was found to be 14.328 h. Previous studies on barberry fruit demonstrated that the air velocity had no major effect on the quality of the dried fruits, whereas the air temperature significantly affected Hunter color values [31]. The same team investigated the dehydration kinetics of barberry at different drying temperatures. They concluded that the use of low temperatures (e.g., 60 °C) is adequate for preserving the color of the dried fruits, and a fruit pre-treatment with olive oil and K_2_CO_3_ reduced the drying time by 40% and 60%, respectively. These results can be further integrated in a study to compare the composition and biological activity of the pretreated fruits versus simple-dried fruits. Another research team investigated the influence of harvesting methods (branch-cutting, cluster picking, and impact force), time of harvest, and drying methods on quality of dried fruits [32]. They concluded that the highest quality of dried fruits was obtained for those that were harvested by the cluster-picking method and shade-dried in late October. These results are in accordance with those obtained by [2]. They documented that the quality of fruit dried in direct sunlight is lower than those dried in mechanical dryers in terms of preserving their natural color and shape. The authors of [33] also concluded in their study that the greatest drying rate in the shortest time (0.1332 kg moisture/kg dry matter) was associated with the samples dried at 55 °C with citric acid pre-treatment.

**Figure 3:**
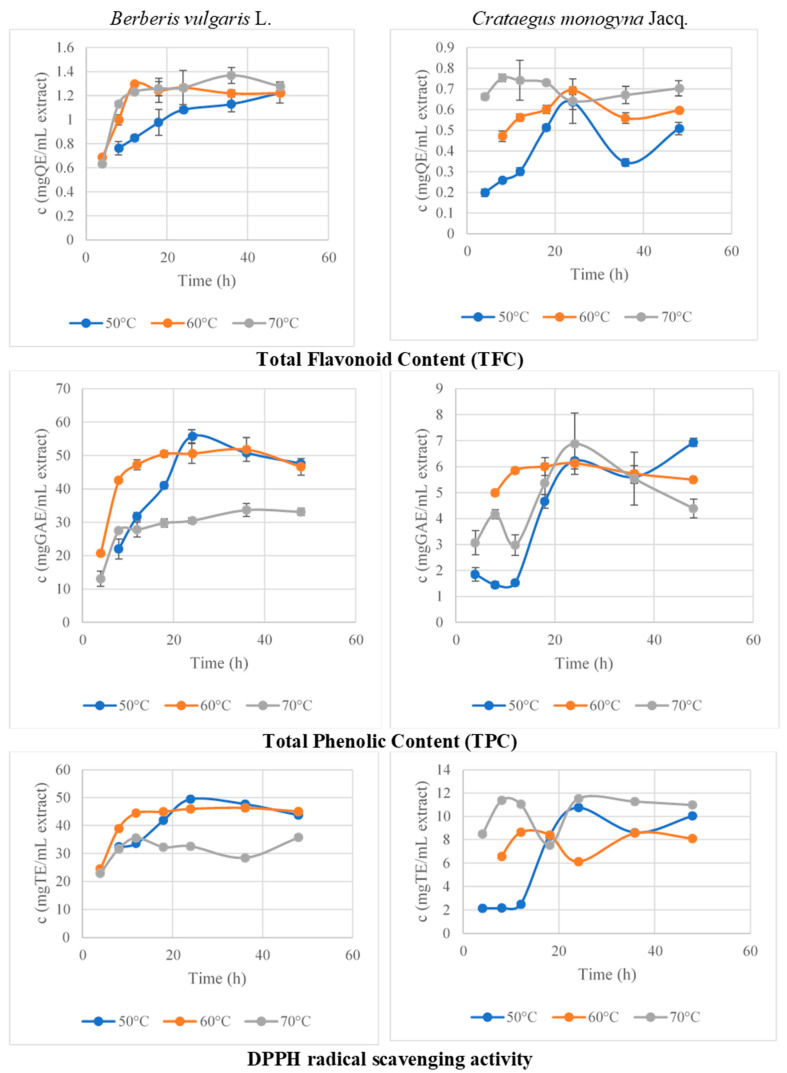
Total phenolic content (TPC), total flavonoid content (TFC), and DPPH radical scavenging activity as a function of time regarding the temperature conditions of drying plant samples (50, 60, and 70 °C).

All these conclusions are in accordance with the results obtained in this study. The samples were evaluated in terms of TFC. As can be seen in Figure 3, the highest amount of flavonoids corresponding to ~14 h of drying was found for the fruits dried at 60 °C. Furthermore, the results from the evaluation of total phenolic content (TPC) of the 14 h —dried berries revealed that those dried at 60 °C were the richest in these compounds (Figure 3) The antioxidant activity can also be correlated with the content of flavonoids and phenolic compounds, with the fruits dried at 60 °C exerting the highest antioxidant capacity (Figure 3). It can be concluded that the ideal temperature for barberry fruit drying is 60 °C. The optimal drying time at 60 °C of *C. monogyna* fruits was 16.1437 h. Koyuncu et al. (2007) evaluated the influence of air temperature on total drying time and total energy requirement [34]. Although the drying parameters were not correlated with any in vivo or in vitro activity of the resultant samples, the authors concluded that the most economic method regarding energy consumption was obtained by drying the fruits at 70 °C. On the other hand, Unal and Sacilik (2011) concluded that the total color change was reduced by decreasing the drying time, and this can be considered a quality parameter of the dried fruits [35]. At this moment, there are not enough data on the correlation between the drying parameters of *C. monogyna* fruits and their biologically active compounds content. The authors of the present study expect that the results presented here may lead to further investigations of the described dried fruits.

According to the preliminary results of the dried fruits after ~16 h of drying, the highest total flavonoid content was obtained for those dried at 70 °C, followed by the fruits dried at 60 °C (Figure 3). On the other hand, the fruits dried at 60 °C exerted the highest total phenolic content (TPC), which correlates with the highest antioxidant capacity of the extracts. These results suggest that the optimal drying conditions are ~16 h of drying at 60 °C air temperature.

### 3.2. Phenolic Compounds Identification Using HPLC/MS

**Figure 4:**
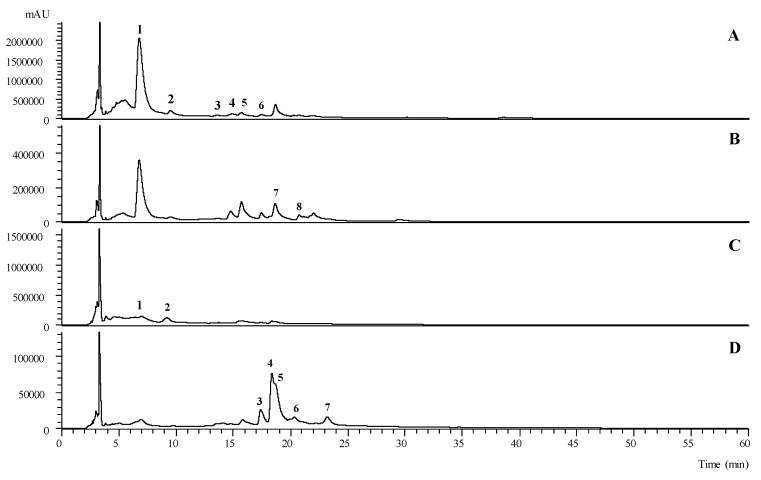
Chromatographic profile of *B. vulgaris* and *C. monogyna* recorded at 280 nm ((**A**) and (**C**), respectively) and 370 nm ((**B**) and (**D**), respectively).

Fifteen phenolic compounds where tentatively identified in the hydroethanolic extracts of *B. vulgaris* (peaks 1 to 8) and *C. monogyna* (peaks 9 to 15) (Table 1): seven flavonoids, five phenolic acids, two flavanonols, and one flavan-3-ol. A phenolic profile of both samples, recorded at 280 nm and 370 nm, is presented in Figure 4.

An *O*-glycosylated derivative of luteolin, peak **7** ([M − H]^−^ at *m*/*z* 593), with an a unique MS^2^ fragment at *m*/*z* 285 (luteolin aglycone), which corresponded to the loss of 176 u, tentatively identified as luteolin-*O*-glucuronide, was also identified in *B. vulgaris* samples. On the other hand, in *C. monogyna* samples a *C*-glycosilated derivative of apigenin was tentatively identified at peak 15, presenting a pseudomolecular ion [M − H]^−^ at *m*/*z* 619 and MS^2^ fragments at *m*/*z* 499, 413, and 393, which was tentatively identified as apigenin 2″-*O*-rhamnosyl-*C*-acetylhexoside based on the description previously given by Barros et al. (2012) in the hydromethanolic extracts of *C. monogyna* from Northeastern Portugal [36].

The second largest family of compounds found was phenolic acids, being predominantly present in *B. vulgaris* samples, mainly as hydroxycinnamic acid derivatives. The fact that *B*. *vulgaris* samples presented a large number and variety of phenolic acids, mainly derived from hydroxycinnamic acids, is consistent with previously described findings by Fernández-Poyatos et al. (2021) on the leaves of *Berberis hispanica* Boiss. & Reut. and Fernández-Poyatos et al. (2019) [37,38] on the leaves of *Berberis thunbergii* DC. It is not surprising that one of these compounds is the major one found in *B. vulgaris* samples, representing 65% of the total amount of found phenolics (peak 1—34.1 ± 0.6 mg/g extract). Peaks 1 and 2 presented a pseudomolecular ion [M − H]^-^ at *m/z* 353, MS^2^ fragments at *m/z* 191, 179, 173, and 135, and a UV-vis spectra maximum of 324/325 nm, consistent with caffeoylquinic acid derivatives. These numbers, elution orders, and fragment abundance are consistent with those previously described by Clifford et al. (2003), leading to their tentative identification as 3-*O*-caffeoylquinic acid (1) and 5-*O*-caffeoylquinic acid (2) [39]. Furthermore, peaks 5 and 6 presented a pseudomolecular ion [M − H]^−^ at *m*/*z* 381 and MS^2^ fragments at *m/z* 293, 219, 203,179, 161, and 135, which is in accordance with the fragment identification performed by Zhang et al. (2007) on *Erigeron breviscapus* extracts [40,41]. Due to a lack of information that could help us to identify the caffeoyl positions, 5 and 6 were tentatively identified as CDOA isomer I and II, respectively. The only phenolic acid compound found in *C. monogyna* was *p*-coumaric acid ([M − H]^−^ at *m*/*z* 163, peak 9), which was identified by comparison of its the retention time, UV-vis spectra, and mass fragmentation with the available standard compound.

The flavonoid family of compounds was the one that stood out the most in *C. monogyna* samples, with five compounds tentatively identified, which did not happen in *B. vulgaris* samples, with only two tentatively identified. *O*-glycosylated quercetin derivatives were the ones found in higher numbers. Peaks 11 ([M − H]^−^ at *m*/*z* 609) and 12 ([M − H]^−^ at *m*/*z* 463) were found to be quercetin-3-*O*-rutinoside and quercetin-3-*O*-glucoside, respectively, by comparing their retention time, UV-vis spectra, and mass fragmentation of available commercial standards. Peak 13 ([M − H]^−^ at *m*/*z* 463), presented the same chromatographic responses as peak 12, except for the retention time, which does not allow us to definitively identify the sugar moiety linked to the quercetin aglycone. It was thus tentatively identified as quercetin-*O*-hexoside. Peak 8 ([M − H]^−^ at *m/z* 447), which presented a unique MS^2^ fragment at *m/z* 301 corresponding to the loss of 146 u (deoxyhexosyl moiety), was tentatively identified as quercetin-*O*-deoxyhexoside. Finally, peak 14 presented a pseudomolecular ion [M − H]^−^ at *m*/*z* 505 and two subsequent MS^2^ fragments at *m/z* 463 (42 u) and *m*/*z* 301 (162 u) due to the loss of acetyl and hexosyl moieties, respectively, and was thus tentatively identified as quercetin-*O*-acetylhexoside.

Two flavanonols were tentatively identified in *B. vulgaris* samples, peaks 3 and 4 ([M − H]^−^ at *m*/*z* 335), that presented a fragmentation MS^2^ pattern consistent with that previously described by Kang et al. (2016) in the hydroethanolic extracts of sorghum wholegrains [42]. Finally, the only flavan-3-ol was identified (in *C. monogyna* samples), peak 10 ([M − H]^−^ at *m/z* 289), by comparison with the available standard compound in terms of retention time, UV-vis spectra, and mass fragmentation. Despite presenting with a lower concentration of total phenolic compounds than the *B. vulgaris* samples, the phenolic profile described for *C. monogyna* is consistent with the existing literature [43,44,45].

### 3.3. Total Phenolic Content (TPC) and Total Flavonoid Content (TFC)

The purpose of this research was to identify the optimum drying conditions for the extraction of the bioactive compounds from plant materials from *B. vulgaris* and *C. monogyna*.

The obtained results are presented in Table 2 and were compared with those from the literature related to bioactive compounds found in the composition of the studied plants’ materials. Furthermore, a correlation between chemical composition and pharmacological effects of fruit extracts was realized in order to underline plants’ utilities.

Flavonoids are secondary plant metabolites with low molecular weights. They are phenolic compounds that can be found in either a free state or a glycosylated state. Due to their nature, the scientific literature documents the presence of a wide range of pharmacological actions with beneficial effects across all body systems (neuroprotective and anti-convulsive effects on the central nervous system, treatment and prevention of some particular cardiovascular diseases, treatment of dyslipidemia, etc.). Recent studies have shown that flavonoids are effective at targeting biological processes that affect the development of type 2 diabetes mellitus, inflammation, and immune system activity [46]. Besides the aforementioned effects, flavonoids pose antitumoral, antimicrobial, and antifungal activities [47].

A spectrophotometric assay based on aluminum-complex formation is one of the most applied methods for the quantification of TFC in food and medicinal plants. Although in the study of Pękal and Pyrzynska (2014) this method showed irregular interaction with all flavonoid compounds, we consider this a preliminary step towards investigating the composition of plants we wish to study in a more in-depth manner using modern techniques [48].

The extract obtained from dried barberry fruit *(B. vulgaris)* exerted a high value in terms of TFC (8.306 ± 0.509 mg QE/g dw extract). As far as we know, the inside of *B. vulgaris* fruits have not been assessed in terms of flavonoid content, so a comparison cannot be made.

Considering the *C. monogyna* extract, the value of 2.584 ± 0.238 mg QE/g dw was similar to that obtained by [49] from *C. monogyna* species collected from the region of Valenciennes, Northern France (2.60–4.90 mg GAE/g dw). In their study, they deep-froze the fresh berries in order to preserve the bioactive constituents. Furthermore, the extraction procedure was slightly different from the one used in our study. The plant material was macerated for 24 h at 4 °C in a 500 mL methanol/water mixture (30/70, *v/v*), followed by the selective extraction of compounds with dichloromethane. The authors of [50] also evaluated the TFC of the *C. monogyna* berries, collected from Chile. They obtained a higher value in terms of total flavonoids (8.77 mgQE/g dw), which can be ascribed to the fact that the pulp of the fruits is richer in flavonoids than the whole pseudofruit. In a recent study, [51] evaluated the total flavonoid content of berries collected from Bragança, Northeastern Portugal. The value obtained varied from 21.70 to 436.34 mg CE/g per extract. A comparison could not be made because of the different standards used. In Sarajevo, Bosnia and Herzegovina, [52] evaluated the flavonoid content of pseudofruits, which exerted 0.254–0.595 mg RUE/g of fresh fruits. Again, a comparison could not be made, due to the different means of result expression.

Secondary metabolites that are derivatives of the pentose phosphate, shikimate, and phenylpropanoid pathways in plants are known as phenolic compounds. Phenolic compounds are widely distributed in plants, playing important roles in their physiology and morphology. Their physiological properties are the main reason why phenolic compounds have been intensively studied in recent years. Among the most important physiological properties are their anti-allergenic, antioxidant, anti-inflammatory, anti-atherogenic, antimicrobial, anti-thrombotic, vasodilatory, and cardioprotective effects [53]. Although this approach has some drawbacks, we used the Folin-Ciocâlteu method to assess total phenolic content in fruits extracts because of its easy application [54].

*B. vulgaris* fruits extract was evaluated in terms of total phenolic content and the value obtained was 100.862 ± 1.967 mg GAE/g dw. Özgen et al. assessed the TPC of fresh fruits collected from Sivas province in Turkey [55]. The samples were processed right after the collecting, and the result ranged between 2565 and 3629 mg GAE per liter of fruit juice. Motalleb et al. obtained similar results for fruits collected from Malaysia (100 and 280 mg GAE/g, for water and methanolic extract, respectively) [56]. They dried the plants at a temperature of 65 °C for three days and extracted the chemical constituents in hot water for 20–30 min, followed by extraction in a water:ethanol mixture (1:10). The procedure was repeated twice, then the thick syrup obtained by evaporation in vacuum conditions was freeze-dried. The authors of [57] evaluated the phenolic content in barberry fruits collected from Khorasan province of Iran and obtained 184.1 and 291.22 mg GAE/g dw for aqueous and alcoholic extracts, respectively. The fruits were dried at room temperature in the dark. These results suggest that the temperature can exert a negative effect on chemical the composition of barberry fruits, especially on phenolic compounds. On the other hand, as stated before, the phenolic content was assessed using Folin-Ciocâlteu method. Barberry fruits are known due to their Vitamin C contents, and it is also known that a high temperature can deteriorate its structure. Thus, the lower TPC content can be ascribed to the limitation of the method used.

The total phenolic content was also evaluated for *C. monogyna* fruit extracts. The value (23.371 ± 1.178 mg GAE/g of dry weight extract) is comparable to those found in the scientific literature. The authors of [58] revealed that the extract of the fruits collected from *C. monogyna* and *C. oxycantha*, from Pomoravlje Province, Serbia contain 35.50 ± 2.48 mg GAE/g of extract. Furthermore, the extract from Chilean hawthorn pseudofruits exerted 28.30 ± 0.02 mg GAE/g of extract. Another team from Tunisia evaluated the phenolic content of hawthorn pulp. They concluded that the content in phenolics ranged between 2.2756 and 14.61 mg GAE/g fw [59]. A similar result was obtained by another team from Bosnia and Herzegovina, who concluded that the extract’s phenolics from hawthorn fruits ranged between 2.01 and 4.60 mg GAE/g of fresh fruit. These results suggest that the drying conditions and extraction method play a huge role in the output of these compounds.

### 3.4. Antioxidant Activity

The DPPH assay is widely used to analyze antioxidant activity of plants’ extracts by measuring the extract’s ability to scavenge DPPH radicals and therefore determine free radical scavenging capacity (Table 2). Extract of fruits of *B. vulgaris* exhibited a high antioxidant capacity against DPPH radicals (50.853 ± 0.246 mg TE/g of lyophilized extract). In a study conducted by Motalleb et al., aqueous and ethanolic extracts of barberry exerted an inhibitory concentration value at which the DPPH radical is scavenged by 50% (IC_50_) of 0.65 mg/mL [56]. Furthermore, the DPPH radical scavenging activity of an extract obtained through an optimized process was able to inhibit 91.15% of radicals [60].

The TEAC assay is also extensively employed to assess total radical scavenging capacity. This assay assesses the ability of antioxidants to scavenge the radical cation ABTS^•+^. The total antioxidant activity of lyophilized extracts of barberry assessed by TEAC was 30.983 ± 0.649 mg TE/g dw (Table 2). Other research groups reported a TEAC value of 8.731 ± 0.185 mmol TE/L for barberry juice and 41.1–49.3 mmol TE/L for fruit juice [55,61].

Regarding *C. monogyna*, the DPPH radical scavenging activity of the lyophilized extract was 34.343 ± 1.025 mg TE/g. However, the scientific literature reports inconsistent results. Therefore, Tadic et al. analyzed the DPPH radical scavenging activity of two hawthorn species [58]. The extracts were obtained by percolation and the IC_50_ was 52.05 µg/mL [58]. Another research group from Chile reported a more potent DPPH radical scavenging activity of hawthorn. Methanolic extract exhibited a total antioxidant activity of 3.61 ± 0.01 µg/m. Regarding TEAC assay, hawthorn exerted a value of 12.677 ± 0.618 mg TE/g lyophilized extract (Table 2).

### 3.5. Cytotoxic and Anti-Inflammatory Activity

Supplementary Table S1 presents the results of the cytotoxic activity of tested extracts based on normal and malignant cell line application. Interestingly, our extracts did not show any cytotoxic activity, either on normal, nor on cancerous cell lines.

In recent years, medical scientific research has focused on finding different pharmacological and botanical treatments for cancerous pathologies. This kind of research is focused on treatments with no side effects. Therefore, natural products are in the view of researchers due to their origin and their tendency to provide no serious side effects. Thus, the extracts of *Berberis* have shown great potential in this direction by reporting antitumor effects.

Hanachi et al. reported that barberry extract induced apoptosis in cancerous hepatic cells in rats [62]. Moreover, the *B. vulgaris* extract inhibited hepatic carcinogenesis in an animal model of disease [63]. The aqueous and ethanolic extracts of barberry also inhibited the proliferation of MCF-7 breast cancer cell line, but they had no effect on MCF10-A human breast epithelial cell line [57].

In a study published in 2008 by Tadic et al., researchers found that hawthorn fruit extract has a dose-dependent effect of reducing rat oedema induced by carrageenan. The extract was obtained by percolation of the *C. monogyna* and *C. oxycantha* fruits with 70% ethanol at room temperature [58].

### 3.6. Inhibition of Fungal and Mammalian α-Glucosidase

**Figure 5:**
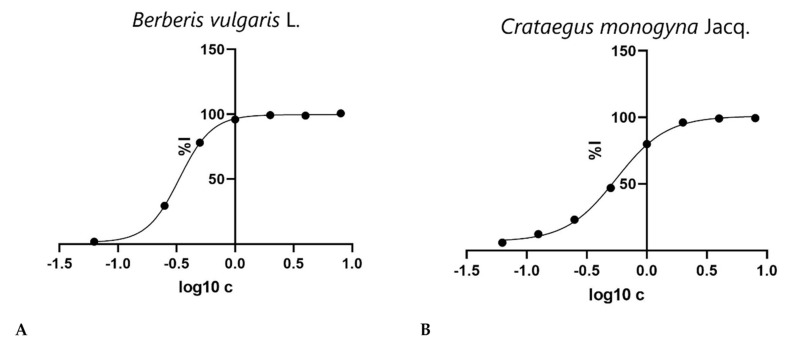
Graphic representation of logarithm of extract concentration (log_10_ [c]) versus the percent of enzyme inhibition (%I) of *B. vulgaris* (**A**) and *C. monogyna* (**B**) extracts.

For dietary carbohydrates to be digested, the presence of α-glucosidase in the small intestine is of particular importance. Inhibitors are molecules that retard carbohydrate digestion, therefore suppressing glucose absorption and postprandial hyperglycemia [64]. Due to the capacity of some natural compounds from plant sources to inhibit α-glucosidase, the importance of the assays examining the relation between compounds’ capacity to inhibit α-glucosidase and enzyme activity is well known. Table 3 presents our study data pertaining to the inhibitory activity of α-glucosidase. Figure 5 presents the logarithm of extract concentration [mg/mL] as a function of %I (percent of enzyme inhibition).

Hajzadeh et al. have shown that the maceration of *Berberis* fruits for 3 h followed by extraction did not have any effect on the lipidic profile of studied animals. Barberry extracts with a concentration of 3.5% and 7.5%, administered for six weeks, presented no hypoglycemic or hypolipidemic effects in rats with streptozotocin induced diabetes [65].

Some in vivo studies have demonstrated that barberry fruit extract has no effect on glucose profile. However, studies have reported inconsistent data. Some researchers have mentioned a beneficial effect of *B. vulgaris* against diabetes and its subsequent pathophysiological effects [65,66]. Moreover, following the beneficial effect of barberry on diabetic markers, researchers were interested in studying the mechanisms by which fruits exert their antidiabetic effect. Two clinical studies identified potential explanations for the aforementioned effect. The first stated that a barberry extract obtained by fruit infusion has a beneficial effect on lipoprotein and apoprotein levels and could control glycemic levels of patients [67]. The second stated that barberry fruit extract could improve glucose catabolism (by supporting aerobe glycolysis), insulin secretion, and its action, but that it may also act on glucose absorption [68].

## 4. Conclusions

The drying of medicinal edible plant materials is a process that consists of various physical, chemical, and biological phenomena. It is very important to understand them in order to achieve high yields of bioactive compounds. In this paper, the drying process of two edible fruits traditionally used as remedies in Romania (i.e., *B. vulgaris* and *C. monogyna*) was optimized using an experimental design. It was found that the optimal time for drying *B. vulgaris* fruits at 60 °C was 14.32 h, whereas that for *C. monogyna* fruits at the same temperature was 16.14 h. Furthermore, the extracts obtained by applying optimal drying conditions yielded high antioxidant activity (barberry fruits—50.85, 30.98, and 302.45 mg TE/g dw for DPPH, TEAC, and FRAP assays, respectively). Moreover, *B. vulgaris* and *C. monogyna* extracts exhibited outstanding α-glucosidase inhibitory activity (IC_50_ = 0.34 and 0.56 mg/mL, respectively). In the light of these results, the main bioactive phenolic compounds, mainly derived from hydroxycinnamic acids, were identified and quantified. Last but not least, industry could benefit from implementing this process, but more studies are needed to deeply understand the phenomena involved in the drying process of studied plant materials.

## Figures and Tables

**Table 1:** The retention time (Rt), wavelengths of maximum absorption (λ_max_), tentative identification, mass spectral data, and quantification of phenolic compounds identified in the extracts of *B. vulgaris* and *C. monogyna* (Mean ± SD).

Peak	Rt (min)	λ Max (nm)	[M − H]^−^ (*m*/*z*)	MS^2^ (*m/z*)	Tentative Identification	Quantification (mg/g Extract)
*B. vulgaris*						
1 ^G^	6.78	324	353	191 (100), 179 (51), 173 (7), 135 (5)	3-*O*-Caffeoylquinic acid	34.1 ± 0.6
2 ^G^	9.51	325	353	191 (100), 179 (11), 173 (5), 135 (5)	5-*O*-Caffeoylquinic acid	3.4 ± 0.1
3 ^F^	14.76	334	335	291 (100), 273 (12), 247 (5), 229 (10), 193 (5), 179 (15), 151 (5)	Hydroxy ampelopsin isomer I	2.3 ± 0.1
4 ^F^	15.71	336	335	291 (100), 273 (12), 247 (5), 229 (10), 193 (5), 179 (15), 151 (5)	Hydroxy ampelopsin isomer II	1.68 ± 0.01
5 ^G^	17.45	332	381	293 (5), 219 (2), 203 (5), 179 (100), 161 (15), 135 (21)	CDOA isomer I	1.5724 ± 0.03
6 ^G^	18.66	328	381	293 (5), 219 (5), 203 (5), 179 (100), 161 (9), 135 (17)	CDOA isomer II	4.55 ± 0.05
7 ^D^	20.75	330	593	285 (100)	Luteolin-*O*-glucuronide	0.558 ± 0.003
8 ^D^	22.02	335	447	301 (100)	Quercetin-*O*-deoxyhexoside	0.61 ± 0.01
					Total phenolic compounds	49 ± 1
*C. monogyna*						
9 ^A^	6.99	310	163	119 (100)	*p*-Coumaric acid	0.1704 ± 0.001
10 ^B^	9.35	280	289	245 (100), 205 (29)	(+)-Catequin	0.78 ± 0.01
11 ^C^	17.39	349	609	301 (100)	Quercetin-3-*O*-rutinoside	tr
12 ^D^	18.37	354	463	301 (100)	Quercetin-3-*O*-glucoside	0.202 ± 0.004
13 ^D^	18.66	352	463	301 (100)	Quercetin-*O*-hexoside	0.194 ± 0.001
14 ^D^	20.33	352	505	463 (100), 301 (25)	Quercetin-*O*-acetylhexoside	0.132 ± 0.001
15 ^E^	23.19	324	619	499 (5), 413 (71), 393 (100)	Apigenin 2″-*O*-rhamnosyl-*C*-acetylhexoside	0.088 ± 0.001
					Total phenolic compounds	1.568 ± 0.002

tr—traces. Standard calibration curves: A—*p*-coumaric acid (*y* = 301950*x* + 6966.7, *R*^2^ = 0.9999, LOD = 0.68 μg/mL; LOQ = 1.61 μg/mL); B—(+)-catequin (*y* = 84950*x* − 23200, *R*^2^ = 1, LOD = 0.17 μg/mL; LOQ = 0.68 μg/mL); C—quercetin 3-*O*-rutinoside (*y* = 13343*x* + 76751, *R*^2^ = 0.9998, LOD = 0.18 μg/mL; LOQ = 0.65 μg/mL); D—quercetin 3-*O*-glucoside (*y* = 34843*x* − 160173, *R*^2^ = 0.9998, LOD 0.21 μg/mL; LOQ 0.71 μg/mL); E—Apigenin-6-*C*-glucoside (*y* = 107025*x* + 61531, *R*^2^ = 0.9989, LOD = 0.19 µg/mL; LOQ = 0.63 µg/mL); F—naringenin (*y* = 18433*x* + 78903, *R*^2^ = 0.9998, LOD = 0.17 µg/mL; LOQ = 0.81 µg/mL); G—chlorogenic acid (*y* = 168823*x* − 161172, *R*^2^ = 0.9999, LOD = 0.20 µg/mL; LOQ = 0.68 µg/mL). CDOA—caffeoyl-2,7-anhydro-3-deoxy-2-octulopyranosonic acids.

**Table 2:** Total phenolic content (TPC), total flavonoid content (TFC), and antioxidant activity.

	*C. monogyna*	*B. vulgaris*
TFC (mg QE/g dw)	2.584 ± 0.238	8.306 ± 0.509
TPC (mg GAE/g dw)	23.371 ± 1.178	100.862 ± 1.967
DPPH (mg TE/g dw)	34.343 ± 1.025	50.853 ± 0.246
TEAC (mg TE/g dw)	12.677 ± 0.618	30.983 ± 0.649
FRAP (mg TE/g dw)	74.341 ± 2.229	302.458 ± 15.257
TBARS (IC_50_; µg/mL)^1^	72.2 ± 0.9	252.5 ± 14.2
OxHLIA Δt = 60 min (IC_50_; µg/mL)^1^	118 ± 7	76 ± 1

**Table 3:** Inhibitory activity against fungal and mammalian α-glucosidase of optimized extracts.

α-Glucosidase Inhibitory Capacity		
Fungal (IC_50_; mg/mL)	0.34 ± 0.01	0.56 ± 0.02
Mammalian (%I; 8 mg/mL)	na	na

na—not active.

## Data Availability

Data are contained within the article or Supplementary Materials.

## Supplementary Materials

The following are available online at www.mdpi.com/article/10.3390/antiox10101579/s1, Table S1: Cytotoxic, anti-inflammatory, and inhibitory activity against fungal and mammalian α-glucosidase of optimized extracts.
