# Supplementary material for: Development of an Optimized Drying Process for the Recovery of Bioactive Compounds from the Autumn Fruits of Berberis vulgaris L. and Crataegus monogyna Jacq
Source: Antioxidants (Basel). 2021 Oct 7;10(10):1579. doi: 10.3390/antiox10101579 (PMC8533465; doi:10.3390/antiox10101579)
Supplement: Supplementary file 1 [file antioxidants-10-01579-s001.zip › antioxidants-1385331-supplementary.pdf]

Supplementary Materials

Supplementary Table S1: Cytotoxic, anti-inflammatory, and inhibitory activity against fungal and mammalian α-glucosidase of optimized extracts.

	B. vulgaris	*C. monogyna*
**Cytotoxic activity** [1] (GI_{50}; μg/mL)		
NCI H460 (non-small cell lung cancer)	>400	>400
MCF-7 (breast carcinoma)	>400	>400
HepG2 (hepatocellular carcinoma)	>400	>400
HeLa (cervical carcinoma)	>400	>400
PLP2 (porcine liver primary culture)	>400	>400
**Anti-inflammatory activity** [2] (IC_{50}; μg/mL)		
NOS production	>400	>400

[1] GI_{50} values for ellipticine 1.03 ± 0.09 μg/mL (NCI-H460), 0.91 ± 0.04 μg/mL (MCF-7), 1.1 ± 0.2 μg/mL (HepG2), 1.91 ± 0.06 μg/mL (HeLa), and 3.2 ± 0.7 μg/mL (PLP2); [2] IC_{50} Dexamethaxone: 16 ± 1 μg/mL (NOS). Different letters in the same row mean significant differences ($p < 0.05$).
